# Comparative Analysis Between Insulated Gel Bags and Direct Cooling for Temperature Management During Kidney Transplant Vascular Anastomosis

**DOI:** 10.3390/jcm14072368

**Published:** 2025-03-29

**Authors:** Yuichi Machida, Tomoaki Iwai, Kazuya Kabei, Junji Uchida

**Affiliations:** Department of Urology, Osaka Metropolitan University Graduate School of Medicine, 1-4-3, Asahi-Machi, Abeno-ku, Osaka 545-8585, Japan; iwai@omu.ac.jp (T.I.); a03ma017@hotmail.co.jp (K.K.); uchida@omu.ac.jp (J.U.)

**Keywords:** kidney transplantation, warm ischemic time, insulated gel bag, renal surface temperature, kidney cooling

## Abstract

**Background/Objectives:** Ischemic time plays a crucial role in graft function and survival during kidney transplantation. Cooling methods, including cold perfusion and ice slush, are predominantly applied to preserve the kidney, but they may cause uneven cooling and complications. The Organ Pocket^®^, an insulated gel bag, has been introduced as an alternative cooling method. However, no studies have compared renal temperature changes between the Organ Pocket^®^ and conventional cooling methods. **Methods:** We retrospectively analyzed 49 cases of living-donor kidney transplantation. Among these, 33 received kidney grafts preserved with the Organ Pocket^®^ (OP group), and 16 underwent conventional cooling (control group). Renal surface temperatures were recorded at 5 min intervals during vascular anastomosis using thermography. Postoperative renal function was assessed with estimated glomerular filtration rate (eGFR), serum creatinine (sCr), and liver-type fatty acid-binding protein (L-FABP) levels. **Results:** The OP group demonstrated significantly higher renal surface temperatures than the control group during vascular anastomosis (*p* < 0.05). Renal surface temperature before reperfusion was 20.4 °C ± 2.5 °C and 17.2 °C ± 2.5 °C in the OP and control groups, respectively. No significant differences in postoperative eGFR, sCr, and L-FABP levels; delayed graft function (DGF); or acute rejection rates were observed between the groups. **Conclusions:** The Organ Pocket^®^ effectively stabilized renal temperatures during vascular anastomosis without direct cooling, thereby reducing continuous manual cooling requirements. Short-term renal function outcomes were comparable between groups; however, the Organ Pocket^®^ may improve surgical efficiency and be particularly beneficial in robot-assisted kidney transplantation. Further studies are warranted to investigate its long-term benefits.

## 1. Introduction

In kidney transplantation, prolonged warm ischemia time (WIT) and cold ischemia time (CIT), along with various donor and recipient factors, are key risk factors for delayed graft function (DGF) and long-term graft survival [1,2]. Diabetes mellitus and prolonged WIT are independent risk factors for poor early graft function [3]. Several studies have reported an association between prolonged WIT and impaired kidney function [4,5,6,7].

As ischemia plays a crucial role in transplant outcomes, kidney cooling is essential to preserve graft function. Hypothermia reduces cellular metabolism, thereby protecting the kidney during ischemia [8]. Renal temperature, which is decreased below the metabolic threshold due to cooling perfusion, increases rapidly during secondary warm ischemia [1,8]. Surface cooling rapidly reduces cortical temperature; however, medullary cooling is delayed, thereby causing a persistent gradient and potential thermal stress on renal tissues. The persistent gradient could disrupt metabolism and increase ischemic injury risk; thus, surface cooling alone may not ensure uniform hypothermia [9,10].

Various cooling methods, including cold perfusion, ice slush bags, ice-soaked gauze, and intra-abdominal cooling systems, are routinely implemented during clinical kidney transplantation to maintain renal temperature and reduce ischemia–reperfusion injury (IRI) and DGF [11,12]. Cold perfusion, which is achieved by flushing the renal vasculature with cold preservation solutions, internally cools the organ but may not maintain low temperatures during prolonged anastomosis [4]. Ice slush bags and ice-soaked gauze, which are predominant external cooling methods, frequently cause uneven temperature distribution, risking inadequate and excessive cooling [5,6]. Prolonged ice slush exposure has been associated with secondary ileus and tissue damage, complicating post-transplant recovery [7,8].

Several new cooling devices have been developed to maintain renal temperature during vascular anastomosis in kidney transplantation, thereby reducing the risk of DGF and IRI. The Net-Patterned Jacket circulates ice water of 0–4 °C around the kidney, thereby stabilizing its temperature at 3.8–5.2 °C and improving graft function in minimal-incision kidney transplantation [13]. Thermal barrier bags insulate the kidney from intraoperative heat, thus slowing temperature elevation during prolonged anastomosis [14]. Intra-abdominal cooling systems, which have been assessed during robot-assisted kidney transplantation, prevent graft rewarming and improve early graft function [12]. These innovations improve temperature control during transplantation and enhance long-term outcomes.

Recently, insulated gel bags were introduced as an alternative for kidney cooling in clinical practice [15]. Ide et al. evaluated the safety and effectiveness of a pouch-type thermal barrier bag (Organ Pocket^®^, Screen Holding Co., Ltd., Kyoto, Japan) made of a transparent biocompatible insulation material specifically designed for kidney transplantation [14].

However, no studies have monitored the temperature changes over time in kidneys cooled using gel bags and those cooled using conventional methods under equivalent secondary warm ischemia conditions.

To address this gap, we measured the temperature changes in kidney grafts using the Organ Pocket^®^, compared them with those caused by conventional direct cooling methods, and assessed their postoperative outcomes.

## 2. Patients and Methods

We retrospectively analyzed 49 cases of living-donor kidney transplantation performed at the Department of Urology, Osaka Metropolitan University, between October 2022 and August 2024. Among them, 33 patients used the Organ Pocket^®^ (OP group), whereas 16 patients did not (control group). Table 1 presents the baseline characteristics of the two groups.

Kidneys were placed in the recipient’s iliac fossa, applying an extraperitoneal approach with vascular anastomosis to the external iliac vessels in all cases. All patients underwent their first renal transplant. Extravesicular ureteroneocystostomy was conducted with a ureteral stent. Intravenous mannitol and methylprednisolone infusion were administered before graft reperfusion. Two experienced transplant surgeons at our institution performed vascular anastomosis in both groups.

All donor kidneys were procured laparoscopically. Immediately after procurement, the grafts were preserved in cooled lactated Ringer’s solution and transported to the recipient-side operating room. On the back table, core cooling was performed using a cooled preservation solution, followed by full perfusion.

In the OP group, the Organ Pocket^®^ was applied after perfusion, wrapped in gauze, and transported to the recipient site for vascular anastomosis. The Organ Pocket^®^ was removed immediately after anastomosis, followed by reperfusion. No cooling solution was introduced into the Organ Pocket^®^ during vascular anastomosis.

In the control group, the renal graft was wrapped in gauze and transported to the recipient site after back-table perfusion. Cold Ringer’s solution was applied to the graft every 2–3 min, as required during vascular anastomosis.

**Figure 1:**
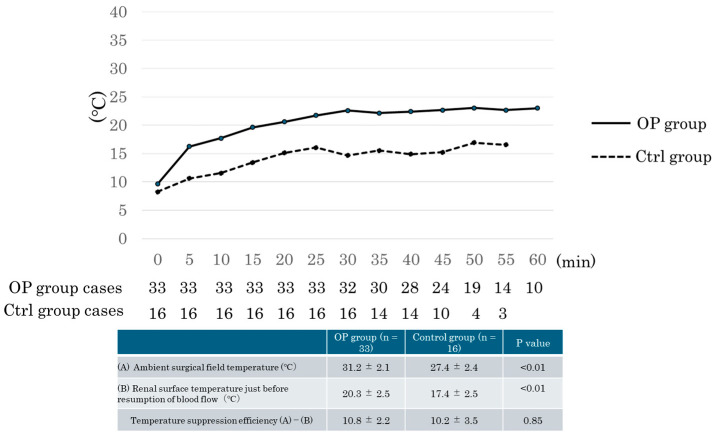
Renal surface temperature changes during vascular anastomosis. This figure compares renal surface temperature changes over time in the Organ Pocket^®^ (OP) group (*n* = 33) and the control (Ctrl) group (*n* = 16). The OP group (solid line) maintained a higher temperature than the Ctrl group (dashed line). The table shows ambient surgical field temperature, renal surface temperature before reperfusion, and temperature suppression efficiency. The OP group had significantly higher temperatures (*p* < 0.01), while suppression efficiency was similar between groups (*p* = 0.85).

In both groups, temperature changes were recorded at 5 min intervals from the start of the vascular anastomosis to the resumption of blood flow using thermography (HIKMICRO EL1, Hikmicro Sensing Technology Co., Ltd., Hikvision, Hangzhou, China). In the OP group, the temperature of the renal hilum was measured as a surrogate for the graft surface temperature while the Organ Pocket^®^ was in place. After the removal of the Organ Pocket^®^, the renal surface temperature was recorded at the end of the vascular anastomosis. Furthermore, the iliopsoas muscle temperature within the surgical field was measured and compared with the renal parenchymal temperature. Figure 1 presents the temperature changes, and Table 2 presents the perioperative outcomes.

Serum creatinine levels were measured using an enzymatic method that is standardized to isotope dilution mass spectrometry. The estimated glomerular filtration rate (eGFR) was calculated using the equation of the Japanese Society of Nephrology as follows: 194 × (age) − 0.287 × (serum creatinine) − 1.094, including a correction factor of 0.739 for women [16]. Urinary L-FABP was measured with an enzyme-linked immunosorbent assay (CIMIC, Tokyo, Japan).

### 2.1. Immunosuppression Protocol

The standard immunosuppressive regimen comprised induction therapy with basiliximab, a calcineurin inhibitor (CNI), everolimus (EVR), and methylprednisolone. For ABO-incompatible kidney transplant recipients, low-dose rituximab (100 mg/m^2^) was administered 2 weeks before the transplantation. Maintenance immunosuppression included CNI, EVR, and low-dose prednisone.

### 2.2. Statistical Analysis

Statistical analyses were performed using EZR (Saitama Medical Center, Jichi Medical University, Saitama, Japan), a graphical user interface for R (The R Foundation for Statistical Computing, Vienna, Austria). EZR is a modified version of R that incorporates commonly used biostatistical functions. Quantitative variables are presented as medians and ranges. Comparisons between groups were performed using Student’s *t*-test, the Wilcoxon–Mann–Whitney test, and the Chi-square test, as appropriate. To assess the normality of data distribution, we conducted the Kolmogorov–Smirnov test. We used a non-parametric test instead of a parametric test to ensure the validity of the statistical analysis if the data did not follow a normal distribution. To address the risk of type I errors from multiple comparisons, we applied the Bonferroni correction. A post hoc power analysis was conducted with GPower to assess the statistical power of our study. The observed effect size (Cohen’s d) was calculated based on the mean differences between the groups and pooled standard deviations. An alpha level of 0.05 and the observed sample size were used to compute the statistical power.

## 3. Results

Table 1 presents the baseline characteristics of the recipients and donors. No significant differences in the recipient or donor age, sex, body mass index (BMI), CIT, or WIT were observed between the OP and control groups.

Figure 1 presents changes in renal surface temperature as measured by thermography. Before the vascular anastomosis, no significant difference in temperature was observed between the two groups (OP group: 9.6 °C ± 3.0 °C; control group: 8.2 °C ± 2.7 °C; *p* = n.s.). However, significant differences in temperature were observed between the two groups at 5, 10, 15, 20, 25, and 30 min after the start of the vascular anastomosis. The renal surface temperatures in the OP group were 16.3 °C ± 3.6 °C, 17.7 °C ± 2.5 °C, 19.6 °C ± 2.7 °C, 20.6 °C ± 2.0 °C, 21.7 °C ± 1.7 °C, and 22.6 °C ± 2.7 °C at these time points, respectively. In the control group, the temperatures were significantly lower at 10.6 °C ± 3.5 °C, 11.6 °C ± 3.5 °C, 13.4 °C ± 3.6 °C, 15.1 °C ± 2.6 °C, 16.1 °C ± 2.8 °C, and 14.7 °C ± 2.6 °C, respectively. Before reperfusion and after the removal of the Organ Pocket^®^, the renal surface temperature was 20.4 °C ± 2.5 °C in the OP group and 17.2 °C ± 2.5 °C in the control group (*p* < 0.05). Similarly, after the vascular anastomosis, the surface temperature before blood flow resumption was significantly higher in the OP group (20.3 °C ± 2.5 °C) than in the Ctrl group (17.4 °C ± 2.5 °C) (*p* < 0.05). However, no significant difference was found between the two groups in terms of temperature differences between the renal parenchyma and surrounding surgical field.

**Figure 2:**
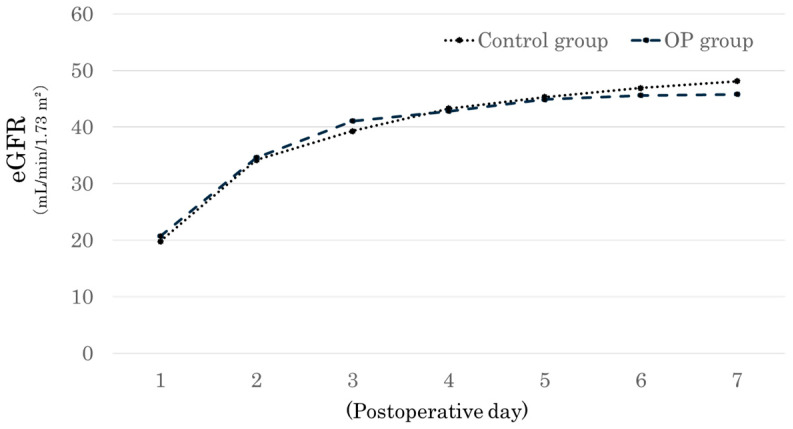
Postoperative changes in eGFR. This figure shows the estimated glomerular filtration rate (eGFR) trends from postoperative day (POD) 1 to POD 7 in the Organ Pocket^®^ (OP) group (dashed line) and the control group (dotted line). Both groups exhibited a similar increase in eGFR over time, with no significant differences between them.

Figure 2 shows the changes in the estimated glomerular filtration rate (eGFR) after transplantation. The eGFR trajectory from postoperative day 1 to day 7 exhibited no significant difference between the two groups. Considering that the baseline characteristics shown in Table 1 were comparable between the two groups, the postoperative renal function recovery was considered equivalent between the two groups.

DGF occurred in one patient who was subsequently diagnosed with antibody-mediated rejection and successfully treated. Furthermore, liver-type fatty acid-binding protein (L-FABP), a biomarker of acute kidney injury, was measured on postoperative days 1, 7, and 28, with no significant difference between the two groups.

The incidence rates of acute rejection during the follow-up period were 18.7% (n = 3) in the control group and 6.1% (n = 2) in the OP group, with no significant difference. Furthermore, no significant differences in serum creatinine levels or eGFR were observed between the two groups during the follow-up period. A post hoc power analysis revealed a statistical power of 0.12, indicating the inefficiency of the study in detecting a clinically meaningful difference. Hence, the non-significant results should be interpreted with caution.

## 4. Discussion

The current gold standard for kidney graft preservation in transplantation is static cold storage at 4 °C [17]. Experimental studies have shown that metabolic activity in the kidney resumes at temperatures between 15 °C and 18 °C after cold storage [18,19]. Therefore, various strategies have been explored to minimize temperature elevation during the secondary warm ischemia time (SWIT) [20,21]. The Organ Pocket^®^ is the first insulated gel bag introduced for clinical use, and this study provides novel insights into renal surface temperature changes during vascular anastomosis. Dergham et al. [21] studied the effect of an insulated cooling jacket on a pig kidney and used thermocouples, revealing that uniform cooling was maintained during a 60 min WIT with no significant difference between surface and core temperatures. This indicates that kidney surface temperature reliably estimates core temperature when using insulated bags such as the Organ Pocket^®^. In our study, we did not measure core temperature with thermocouples due to their invasive nature. However, we consider thermography a similarly practical and noninvasive method for assessing renal temperature dynamics. However, intermittent cooling rapidly decreased surface temperature immediately after application in the control group, likely causing a discrepancy between the actual kidney surface and core temperatures.

Previous reports have demonstrated the temperature-suppressing effect of the Organ Pocket^®^ [14]; however, this study is the first to compare a group in which cooling fluid was intermittently applied to the graft during vascular anastomosis with a group in which no direct cooling was administered. Our results showed a significantly higher renal surface temperature in the OP group than in the control group during anastomosis. However, because thermographic measurements were taken at the renal hilum, which was not in direct contact with the Organ Pocket^®^, the recorded temperature may have been influenced by surrounding tissue. Nonetheless, after the removal of the Organ Pocket^®^ and before reperfusion, the renal surface temperature stabilized at approximately 20 °C, demonstrating effective temperature control. Importantly, no significant differences in ambient temperature control were observed between the two groups.

Kuipers et al. examined renal temperature changes during vascular anastomosis without an insulated bag in living-donor kidney transplantation. Using thermography, they measured renal temperatures after wrapping a wet cooling sponge around the kidney and reported temperatures of 13.7 °C, 17.4 °C, and 20.2 °C at 10, 20, and 30 min, respectively [1]. In contrast, the control group in our study exhibited lower temperatures, likely due to the intermittent application of cooling fluid. Although thermographic renal surface temperature measurement is a noninvasive and clinically feasible method, assessing core renal temperatures using thermocouples may provide more accurate evaluations. However, because of the invasiveness of such a procedure, surface temperatures remain a practical surrogate.

Prolonged SWIT is correlated with poorer graft function, emphasizing the importance of minimizing ischemic time [2,22,23]. In our study, apart from one case of antibody-mediated rejection, no cases of DGF were observed. The use of an insulated gel bag, the Organ Pocket^®^, effectively prevented temperature increases of >10 °C without requiring direct cooling during vascular anastomosis. Furthermore, the absence of significant differences in liver-type fatty acid binding protein (L-FABP), a biomarker of acute kidney injury [24,25], between the two groups suggests that Organ Pocket^®^ use did not compromise short-term graft function.

Another potential advantage of insulated gel bags is their applicability in robot-assisted kidney transplantation (RAKT). Although RAKT has not yet been widely adopted in Japan, it is increasingly being used worldwide [26,27]. This approach offers several benefits, such as reduced surgical site infection risk, improved graft function, and superior patient outcomes, particularly in individuals with obesity [28]. However, because vascular anastomosis is performed intraperitoneally using robotic assistance, traditional cooling methods involving direct fluid application may interfere with the procedure, affect intra-abdominal organs, and prolong anastomosis time, potentially compromising graft function. The use of an insulated gel bag in RAKT could help mitigate these issues by effectively preventing temperature elevation during anastomosis, thereby promoting early graft function recovery.

The Organ Pocket^®^ provides practical advantages in clinical implementation and requires no large-scale equipment installation or specialized additional training, making it relatively cost-effective compared with other potential solutions. Traditional cooling methods often need frequent cooling fluid application and manual temperature adjustments, which can be labor-intensive. Conversely, the Organ Pocket^®^ may reduce the need for continuous manual cooling, thereby improving surgical efficiency. An associated product cost exists, but its ease of implementation and procedural simplicity made it an economically viable option. However, objective data on surgical duration, staff workload, and recipient hypothermia risk were not obtained, making its potential benefits hypothetical. Further research is warranted to confirm these effects.

At present, the Organ Pocket^®^ is the only commercially available product designed for this purpose, and similar products remain in development. Its ease of use and efficiency in maintaining kidney graft temperature during transplantation emphasize its potential clinical use. Future research is recommended to assess its applicability in diverse surgical settings, including its use in robotic-assisted procedures.

This study has several limitations. This retrospective single-center study carries a risk of selection bias and limited generalizability. Despite comparable baseline characteristics, unmeasured confounders may have affected the results, and the small sample size precluded bias-reduction methods such as propensity score matching. Further, key confounders, including age, CIT, WIT, BMI, and ABO compatibility, were not adjusted for. Multivariable regression was not conducted to prevent overfitting, and the observed effect size was small (Cohen’s d = 0.24). Post hoc analysis revealed low statistical power (0.12), indicating that the study was underpowered. Larger, multi-center studies are warranted for validation and more robust conclusions. The lack of a long-term follow-up limits graft survival assessment, as short-term renal function alone is insufficient. Future studies should assess long-term outcomes at 1 and 5 years. Moreover, the immunological impact of cooling was not assessed, and biomarkers, such as interleukin-6 and tumor necrosis factor-α, should be investigated. Furthermore, the safety of the Organ Pocket^®^ was not assessed, and potential complications, including infections or technical issues, require further investigation.

## 5. Conclusions

This study demonstrated that the Organ Pocket^®^ effectively suppressed renal temperature elevation during vascular anastomosis without requiring direct cooling. Postoperative renal function outcomes, including eGFR and L-FABP levels, were comparable between the two groups, with no significant differences in acute rejection or DGF. Although core temperature measurements were not performed, the thermographic analysis provided valuable insights into temperature control. The Organ Pocket^®^ may reduce the surgical burden and improve the consistency of cooling, particularly for RAKT. Further studies are necessary to confirm the long-term benefits associated with the Organ Pocket^®^.

## Figures and Tables

**Table 1:** Baseline characteristics of recipients and donors in the control and OP groups.

	Control (*n* = 16)	Organ Pocket (*n* = 33)	*p*-Value
Recipient age	44 (34.75–54)	46 (40–54)	0.798
Recipient sex	8/8	19/14	0.718
Recipient BMI (kg/m^2^)	22.6 (17.9–25.9)	23.2 (23.2–26.4)	0.466
Harvested kidney volume (mL)	130.8 (125.4–147.6)	142.5 (123.4–163.7)	0.43
Donor age	60.5 (54.5–62.5)	55 (49–64)	0.55
Donor sex	5/11	13/20	0.754
Donor BMI (kg/m^2^)	23.1 (21.3–24.2)	22.2 (20.7–25.3)	0.647
Operation time (min)	294.0 (250.75–332.75)	290.5 (237.5–336.0)	0.913
CIT (min)	22.5 (16.5–24.75)	22.5 (19.75–27)	0.298
WIT (min)	3 (2–3)	3 (3–3)	0.281
SWIT (min)	51 (45–54)	55 (48.5–64.75)	0.123
ABO compatible	Compatible: 14 Incompatible: 2	Compatible: 22 Incompatible: 11	0.174
Number of renal arteries	One: 14 Two: 2	One: 26 Two: 7	0.698

This table presents baseline characteristics of recipients and donors in the control (*n* = 16) and Organ Pocket^®^ (*n* = 33) groups, including age, sex, BMI, kidney volume, CIT, WIT, SWIT, ABO compatibility, and renal artery count. No significant differences were observed between groups. Data are shown as median (IQR) or count.

**Table 2:** Postoperative outcomes between the control and OP groups.

	Control (*n* = 16)	Organ Pocket (*n* = 33)	*p*-Value
Postoperative period (month)	11.5 (10–18)	8 (4–24)	0.515
Best sCr	0.94 (0.8–1.41)	1.03 (0.92–1.3)	0.533
Best eGFR	52.2 (45.6–69)	50.6 (45.1–63.1)	0.689
Delayed graft function	1	0	0.327
L-FABP POD1	92.4 (59.8–138.5)	88.1 (44.2–170)	0.727
L-FABP POD7	19.1 (4.3–60.9)	29.3 (6.7–81)	0.564
L-FABP POD28	22.3 (5.7–22.3)	6.8 (4.3–12)	0.678
Acute rejection	3	2	0.163

This table compares postoperative outcomes between the control (*n* = 16) and Organ Pocket^®^ (*n* = 33) groups. Parameters include follow-up duration, best serum creatinine (sCr), best estimated glomerular filtration rate (eGFR), delayed graft function (DGF), acute rejection, and liver-type fatty acid-binding protein (L-FABP) levels on postoperative days (POD) 1, 7, and 28. No significant differences were observed between groups. Data are presented as median (interquartile range) or count.

## Data Availability

All data generated or analyzed during this study have been included in this published article.

## Abbreviations

WIT	Warm ischemia time
CIT	Cold ischemia time
DGF	Delayed graft function
CNI	Calcineurin inhibitor
EVR	Everolimus
eGFR	Estimated glomerular filtration rate
